# Synthesis and Characterization of Functional Cellulose–Ether-Based PCL- and PLA-Grafts-Copolymers

**DOI:** 10.3390/polym15020455

**Published:** 2023-01-15

**Authors:** Korbinian Sommer, Daniel Van Opdenbosch, Cordt Zollfrank

**Affiliations:** Chair for Biogenic Polymers, TUM Campus Straubing for Biotechnology and Sustainability, Technical University of Munich, 94315 Straubing, Germany

**Keywords:** graft copolymers, cellulose, cellulose ether, poly(ε-caprolactone), poly(L-lactic acid)

## Abstract

The use of biodegradable materials such as cellulose and polyesters can be extended through the combination, as well as modification, of these biopolymers. By controlling the molecular structure and composition of copolymers of these components, it should also be possible to tailor their material properties. We hereby report on the synthesis and characterization of cellulose-based graft copolymers with a precise molecular composition and copolymer architecture. To prepare such materials, we initially modified cellulose through the regioselective protection of the 6-OH group using trityl chloride. The 6-O protected compound was then alkylated, and deprotection at the 6-OH group provided the desired 2,3-di-O-alkyl cellulose compounds that were used as macroinitiators for ring opening polymerization. Regioselective modification was hereby necessary to obtain compounds with an exact molecular composition. Ring opening polymerization, catalyzed by Sn(Oct)_2_, at the primary 6-OH group of the cellulose macroinitiator, using L-lactide or ε-caprolactone, resulted in graft copolymers with the desired functionalization pattern. The materials were characterized using Fourier-transform infrared spectroscopy, ^1^H- and ^13^C- nuclear magnetic resonance spectroscopy, size exclusion chromatography as well as X-ray diffraction, and differential scanning calorimetry. PCL-based copolymers exhibited distinct melting point as well as a crystalline phase of up to 47%, while copolymers with PLA segments were highly amorphous, showing a broad amorphous reflex in the XRD spectra, and no melting or crystallization points were discernible using differential scanning calorimetry.

## 1. Introduction

The preparation of tailored polymers and copolymers that exhibit certain properties is of high relevance for various applications, such as tissue and bone engineering, controlled drug release, as well as other fields that require biocompatible or biodegradable functional materials [1]. Both cellulose [2] and polyesters [3] have found applications in these areas, and the combination of both materials is promising for the preparation of high precision copolymers. The approach for the synthesis of cellulose-based copolymers presented here allows for targeted adjustment of graft density and sidechain length through the initial modification of cellulose, in contrast to grafting reactions with native or randomly substituted cellulose. These initial functionalizations let us control the resulting copolymer composition, which is of great importance for material properties [4].

Poly(caprolactone) (PCL) and poly(lactic acid) (PLA) are aliphatic polymers that can be produced from sustainable resources and exhibit biodegradable properties, while cellulose represents the most abundant biopolymer [5,6]. Both polymers already show potential in numerous applications [7] due to their respective attributes as well as the possibility to functionalize and introduce new reactive sites [8]. Functional polymers and copolymers of polyesters, polycaprolactone in particular, have been reported by direct modification of the respective monomer. Copolymers of α-allylcaprolactone and caprolactone were prepared by Darcos et al. via ring opening polymerization of the two monomers [9]. These compounds were further utilized in thiol-ene reactions with 2-(boc-amino)ethanethiol as reactant. Copolymers of allyl valerolactone and caprolactone were previously prepared by Parrish et al. [10]. Such functionalized copolymers have been reported for the purpose of stabilizing proteins [11,12].

With respect to global environmental sustainability, cellulose-based materials hold great promise due to the abundance of cellulose as a natural resource [13]. Another advantage of cellulose lies in the hydroxyl groups at C2, C3 and C6 that allow the modification and functionalization of the native polymer to introduce new functions and properties. Modifications with various functional groups [13,14], as well as substitution patterns of cellulose, have been reported previously [15,16,17], also with the intention of determining the structure–property relationships of the prepared compounds. Propyl cellulose, for instance, shows tremendous differences in its flocculation temperature through variation of the degree of substitution or the substitution site as analyzed in detail by Heinze et al. [18]. As the mechanical properties of cellulose are strongly dependent on the manifestation of a hydrogen bond network between the hydroxyl groups of the anhydroglucose units, targeted derivatization of cellulose enables the tuning of properties of the resulting compounds and copolymers [19].

Only few advances towards the synthesis of regioselectively substituted copolymers of cellulose have been reported to date in the literature. One approach for the preparation of regiocontrolled cellulose–PCL graft-copoylmers was reported by Wang et al. In their research, an aqueous zinc chloride solution was used as solvent for cellulose which favored grafting of PCL at OH-2 and OH-3 of the anhydroglucose units. PCL was thereby “grafted from” secondary hydroxyl groups of the AGU [20]. Recently, another approach for the synthesis of regioselectively substituted polysaccharide-based copolymers was published. In this work, Tsuji et al. also employed the hydroxyl groups at C2 and C3 of the anhydroglucose unit for the polymerization reaction of ε-caprolactone, while polystyrene was grafted subsequently to the hydroxyl group at C6 via click chemistry for the production of cellulose-based Janus bottlebrushes [21]. Alternatively, copolymers were prepared by a “grafting to” approach using methyl-polyethyleneglycol iodide in a nucleophilic substitution in order to obtain the C2 and C3-substituted cellulose derivative [22], which can also be further modified using grafting reactions, e.g., via click reactions with modified polystyrene as mentioned above [23]. Regioselective grafting to the hydroxyl group at C3 has also been demonstrated by Kadla et al. in their synthesis of silylated cellulose derivatives for the preparation of materials that allow the production of honeycomb patterned films [24,25].

Advances by other authors towards the preparation of polysaccharide-based graft copolymers without a special focus on regioselectivity include the works of Tian et al., and Wohlhauser et al., who grafted PCL onto cellulose nanocrystals using a “grafting from” approach, and verified their successful reaction via infrared spectroscopy [26,27]. Further research in that regard was undertaken using direct grafting of PCL to cellulose pulp in ionic liquids [28]. Using cellulose derivatives for grafting reactions, commercial ethyl cellulose with a DS of 2.3 was utilized in the preparation of cellulose–PCL–PLA grafts copolymers. The authors used commercially available ethyl cellulose, performing the polymerization of PCL in xylene, while the consecutive polymerization of the lactide monomer using the hydroxyl terminated PCL side chains as initiators was performed in bulk. They also reported on the degradation of these compounds and observed a faster degradation when compared to linear PCL [29,30]. Most recently, there has been an effort by Benahmed et al. in their preparation of cellulose acetate-based poly(caprolactone) copolymers [31]. In their research, they pursued a “grafting to” approach using methacrylate modified polycaprolactone and a diisocyanate linker for covalent coupling to the polysaccharide chain similar to previous reports for the modification of cellulose using aliphatic polyesters [32]. Starch-based copolymers with PCL sidechains have also been reported, which were synthesized via ring opening polymerization of ε-caprolactone using ionic liquid catalysts. This was carried out to improve applicability of starch-based materials, and enhance material properties due to the decreased water uptake through combination with hydrophobic PCL [33,34]. Amphiphilic polycaprolactone graft copolymers can also be prepared using chitin derivatives, as in the synthesis of polycaprolactone graft chitosan by Yu et al. [35]. These syntheses also imply a certain degree of regioselectivity as the hydroxyl groups at position 6 were protected by trityl groups and coupling of polyesters was achieved via the amine group of the glucosamine repeating unit and a carbonyl diimidazole activated polycaprolactone.

For this work, we hypothesize that cellulose–backbone graft copolymers would exhibit thermal properties, such as melting points, that are traceable to the thermoplastic component, i.e., the PCL or PLA sidechains in the copolymers, while it is possible to impose a copolymer architecture through initial regioselective modification of the cellulose macroinitiators.

Therefore, for our research, we prepared regioselectively modified cellulose derivatives bearing functional groups, such as benzyl-, allyl-, and propyl moieties at OH-2 and OH-3 of the AGU, to demonstrate the versatility of the cellulose biopolymer in regards to the functionalities that may be introduced into polysaccharide-based copolymers through targeted modification and regioselective reactions, as in our study. After introduction of functionality, the physical properties of these cellulose-based macroinitiators can be further enhanced through the homogeneous ring opening polymerization of L-lactide or ε-caprolactone via the unprotected hydroxyl group at C6 in a “grafting from” approach to prepare the respective copolymers that exhibit a precise and controllable structural composition.

## 2. Materials and Methods

L-lactide (449210000, Acros Organics, Geel, Belgium), ε-caprolactone (802801 Merck, Darmstadt, Germany), cellulose (435236, Merck, Darmstadt, Germany), lithium chloride (62480, Sigma Aldrich, Steinheim, Germany), trityl chloride (93000, Sigma Aldrich, Steinheim, Germany), pyridine (270970, Sigma Aldrich, Steinheim, Germany), *N*,*N*-dimethylacetamide (DMAc, 803235, Merck, Darmstadt, Germany), tetrahydrofuran (THF, 401757, Sigma Aldrich, Steinheim, Germany), toluene (601-021-00-3, VWR, Darmstadt, Germany), sodium hydride (814552, Merck, Darmstadt, Germany) tin(II) 2-ethylhexanoate (Sn(Oct)_2_, AB106427, ABCR, Karlsruhe, Germany) were used in the experiments as received unless otherwise stated. THF and toluene were distilled under inert gas using standard Schlenk techniques and stored over molecular sieves (4 Å) before use. Sn(Oct)_2_ and ε-caprolactone were distilled under reduced pressure in accordance with the procedure reported by Lee et al. [36], and stored under argon atmosphere.

### 2.1. Syntheses

6-*O*-Trityl-cellulose (**2**)

Cellulose (2.0 g, 12.3 mmol) was dissolved in DMAc/LiCl (7%) solvent system as previously reported [37]. Pyridine (10 mL) and triphenyl methylchloride (9.3 g, 33.0 mmol, 3 eq), were added and the solution was stirred (Hei-Connect, Heidolph Instruments, Germany) at 90 °C for 24 h. The product was precipitated, washed with isopropyl alcohol and dried through rotary evaporation to obtain the product as a light beige solid (65 %, 3.2 g, 7.8 mmol).

^1^H NMR (400 MHz, DMSO-*d*_6_) δ 7.71–6.77 (m, H_aromat_), 5.16–2.80 (m, H_cellulose_).

^13^C NMR (101 MHz, DMSO-*d*_6_) δ 144.2 (C_aromat_), 128.9 (C_aromat_), 128.3 (C_aromat_), 127.4 (C_aromat_), 101.1 (C1), 86.15 (-*C*Ph_3_), 76.5–73.9 (C2–C5), 62.4 (C6).

IR [cm^−1^] 3463, 3058, 2883, 1597, 1490, 1029, 899, 763, 746, 699, 643, 632.

6-*O*-trityl-2,3-*di*-O-alkyl-cellulose

6-O-Trityl cellulose (1 eq) was dissolved in dimethylformamide (DMF), and sodium hydride (6 eq) was slowly added. The mixture was heated to 50 °C using an oil bath on a stirring plate, and the alkyl halide (6 eq) was added dropwise. The mixture was then mixed using magnetic stirring for 16 h at 50 °C. The products were precipitated in isopropyl alcohol, washed, and dried using a rotary evaporator.

6-*O*-trityl-2,3-*di*-O-allyl-cellulose (**3**)

The product was obtained as a light brown solid (83%, 2.00 g, 4.11 mmol).

^1^H NMR (400 MHz, Chloroform-*d*) δ 7.68–6.95 (m, H_aromat_), 5.96–5.53 (m, H_allyl_), 5.23–4.65 (m, H_allyl_), 4.47–2.55 (m, H_cellulose_, -C*H*_2_).

^13^C NMR (101 MHz, Chloroform-*d*) δ 143.6 (C_aromat_), 136.7 (C_allyl_), 135.8 (C_allyl_), 129.3 (C_aromat_), 127.9 (C_aromat_), 127.1 (C_aromat_), 115.6 (C_allyl_), 115.2 (C_allyl_), 100.2 (C1), 86.3 (-CPh_3_), 82.4 (C3), 81.7 (C2), 74.5 (C5), 73.9 (C4), 72.7(-*C*H_2_), 61.1 (C6).

IR [cm^−1^] 3060, 2876, 1646, 1597, 1490, 1448, 1419, 1345, 1315, 1220, 1073, 1045, 996, 919, 764, 746, 737, 700, 644, 631, 531.

M_n_ = 5.469 g mol^−1^, M_w_ = 6.723 g mol^−1^, D = 1.2

6-*O*-trityl-2,3-*di*-O-benzyl-cellulose (**4**)

Compound 4 was obtained as a light beige solid (73%, 2.65 g, 4.52 mmol).

^1^H NMR (400 MHz, Chloroform-*d*) δ 7.71–6.80 (m, H_aromat_), 5.16–2.80 (m, H_cellulose_, -C*H*_2_).

^13^C NMR (101 MHz, Chloroform-*d*) δ 143.4 (C_aromat_), 139.4 (C_aromat_), 138.9 (C_aromat_), 129.1–127.1 (C_aromat_), 100.6 (C1), 86.4 (-*C*Ph_3_), 82.8–81.7 (C2, C3), 75.2–74.0 (C4, C5, -*C*H_2_), 61.3 (C6).

IR [cm^−1^] 3060, 3029, 2878, 1598, 1493, 1448, 1359, 1316, 1208, 1153, 1070, 1042, 1028, 1001, 899, 844, 733, 695, 631, 552, 460.

M_n_ = 5.015 g mol^−1^, M_w_ = 6.323 g mol^−1^, D = 1.3

6-O-trityl-2,3-*di*-O-propyl-cellulose (**5**)

The product was isolated as an off-white solid (64%, 3.1 g, 6.32 mmol).

^1^H NMR (400 MHz, Chloroform-*d*) δ 7.76–6.87 (m, H_aromat_), 4.60–2.53 (m, H_cellulose_, -C*H*_2_CH_2_CH_3_), 1.44–1.11 (m, -CH_2_C*H*_2_CH_3_), 0.81–0.40 (m, -CH_2_CH_2_C*H*_3_).

^13^C NMR (101 MHz, Chloroform-*d*) δ 143.6 (C_aromat_), 129.3 (C_aromat_), 127.8 (C_aromat_), 127.0 (C_aromat_), 100.3 (C1), 86.1 (-CPh_3_), 83.1, 82.9, 75.6, 73.8, 72.0 (C2–C5, -*C*H_2_CH_2_CH_3_), 61.2 (C6), 23.5 (-CH_2_*C*H_2_CH_3_), 10.9 (-CH_2_CH_2_*C*H_3_).

IR [cm^−1^] 3059, 2960, 2876, 2933, 1598, 1490, 1448, 1361, 1316, 1218, 1152, 1084, 1040, 1001, 945, 898, 762, 746, 698, 644, 631, 563.

M_n_ = 7.424 g mol^−1^, M_w_ = 11.152, D = 1.5

2,3-*di*-O-alkyl-cellulose

6-*O*-Trityl-2,3-*di*-O-alkyl-cellulose (**3**, **4**, **5**) was dissolved in CHCl_3_ and cooled to 0 °C. Hydrogen chloride gas was then bubbled through the solution under vigorous stirring for 3 min. The deprotected products were precipitated in n-hexane, washed, and dried in vacuo as detailed above.

2,3-*di*-O-allyl-cellulose (**6**)

Compound 6 was obtained as a light brown solid (88%, 0.89 g, 3.64 mmol).

^1^H NMR (400 MHz, Chloroform-*d*) δ 6.07–5.75 (m, H_allyl_), 5.33–4.98 (m, H_allyl_), 4.49–2.98 (m, H_cellulose_, -C*H*_2_).

^13^C NMR (101 MHz, Chloroform-*d*) δ 135.9 (C_allyl_), 134.9 (C_allyl_), 116.7 (C_allyl_), 115.6 (C_allyl_), 102.9 (C1), 82.5 (C3), 81.5 (C2), 77.8 (C5) 75.1 (C4), 74.07 (-C*H*_2_), 73.9 (-C*H*_2_), 60.9 (C6).

IR [cm^−1^] 3406, 3080, 2874, 1733, 1646, 1458, 1420, 1348, 1058, 996, 922, 559.

M_n_ = 3.107 g mol^−1^, M_w_ = 5.852 g mol^−1^, D = 1.8

2,3-*di*-O-benzyl-cellulose (**7**)

The product was obtained as a colorless solid in 81% (0.95 g, 2.76 mmol) yield.

^1^H NMR (400 MHz, Chloroform-*d*) δ 7.46–7.14 (m, H_aromat_), 5.17–3.14 (m, H_cellulose_, -C*H*_2_Ph).

^13^C NMR (101 MHz, Chloroform-*d*) δ 139.3 (C_aromat_), 138.3 (C_aromat_), 128.5–126.8 (C_aromat_), 102.8 (C1), 82.8 (C3), 82,0 (C2), 75.3 (C5, -C*H*_2_Ph), 73.1 (C4), 60.7 (C6).

IR [cm^−1^] 3430, 3063, 2873, 1496, 1453, 1359, 1209, 1060, 1027, 910, 733, 695, 619, 555, 460.

M_n_ = 3.593 g mol^−1^, M_w_ = 5.106 g mol^−1^, D = 1.4

2,3-*di*-O-propyl-cellulose (**8**)

After deprotection, compound **8** was obtained as a light beige solid (81%, 1.24 g, 4.99 mmol).

^1^H NMR (400 MHz, DMSO-*d*_6_) δ 4.96–2.57 (m, H_cellulose_, -CH_2_), 1.40 (s, CH_2_), 0.76 (s, CH_3_).

^13^C NMR (101 MHz, DMSO-*d*_6_) δ 101.9 (C1), 83.1–74.0 (C2-C5, -O*C*H_2_CH_2_CH_3_), 60.3 (C6), 23.47 (-OCH_2_*C*H_2_CH_3_), 11.0 (-OCH_2_CH_2_*C*H_3_).

IR [cm^−1^] 3410, 2962, 2934, 2877, 1462, 1363, 1032, 950, 896, 578.

M_n_ = 4174 g mol^−1^, M_w_ = 9721 g mol^−1^, D = 2.3

6-*O*-PCL-2,3-*di*-O-alkyl-cellulose

Cellulose ethers were mixed with ε-caprolactone in a Schlenk flask and heated to 110 °C in an oil bath using a magnetic stirrer until a clear homogeneous melt was achieved. The polymerization was started by the addition of Sn(Oct)_2_ (0.05 eq), and the reaction mixture was stirred under argon for 16 h at 110 °C. The mixture was dissolved in DCM and precipitated in hexane. The polymer was washed with hexane and a hexane/toluene mixture (20 mL/80 mL), and dried under reduced pressure using rotary evaporation.

6-*O*-PCL-2,3-*di*-O-benzyl-cellulose (**9**)

^1^H NMR (400 MHz, Chloroform-*d*) δ 7.33–7.05 (m, H_aromat_), 5.05–3.16 (m, H_cellulose_, -C*H*_2_Ph), 2.27 (t, *J* = 7.5 Hz, C*H*_2_), 2.27 (t, *J* = 7.5 Hz, C*H*_2_), 1.61 (m, C*H*_2_), 1.35 (m, C*H*_2_).

^13^C NMR (101 MHz, CDCl_3_) δ 173.6 (C=O), 139.0 (C*H*_2_Ph), 138.3 (C*H*_2_Ph), 128.4–127.7 (C_aromat_), 102.8 (C1), 82.7 (C3), 81.9 (C2), 75.0 (C5, -C*H*_2_Ph), 73.0 (C4), 64.2 (C*H*_2_), 34.2 (C*H*_2_), 28.4 (C*H*_2_), 25.6 (C*H*_2_), 24.6 (C*H*_2_).

IR [cm^−1^] 3443, 3031, 2942, 2865, 1722, 1497, 1455, 1418, 1397, 1365, 1294, 1241, 1188, 1162, 1087, 1064, 1045, 961, 933, 840, 733, 698 583, 453.

M_n_ = 19.135 g mol^−1^, M_w_ = 37.108 g mol^−1^, D = 1.9

6-*O*-PCL-2,3-*di*-O-allyl-cellulose (**10**)

^1^H NMR (400 MHz, Chloroform-*d*) δ 5.94–5.71 (m, H_allyl_), 5.26–4.93 (m, H_allyl_), 4.50–2.95 (m,H_cellulose_, -C*H*_2_CHCH_2_), 4.01 (t, *J* = 6.7 Hz, 8H), 2.26 (t, *J* = 7.5 Hz, 9H), 1.60 (m, C*H*_2_), 1.41–1.29 (m, CH_2_).

^13^C NMR (101 MHz, Chloroform-*d*) δ 173.6 (C=O), 135.7 (C=C), 134.8 (C=C), 116.5 (C=C), 115.9 (C=C), 102.8 (C1), 82.5 (C3), 81.4 (C2), 78.3 (C5), 73.81 (-*C*H_2_), 73.1 (C4), 64.2 (-*C*H_2_), 34.2 (-*C*H_2_), 28.4 (-*C*H_2_), 25.6 (-*C*H_2_), 24.6 (-*C*H_2_).

IR [cm^−1^] 3441, 2944, 2865, 1721, 1470, 1419, 1397, 1366, 1294, 1187, 1106, 1065, 1045, 961, 932, 840, 772, 731, 710.

M_n_ = 25.000 g mol^−1^, M_w_ = 45.683 g mol^−1^, D = 1.8

6-*O*-PCL-2,3-*di*-O-propyl-cellulose (**11**)

^1^H NMR (400 MHz, Chloroform-*d*) δ 4.00 (t, *J* = 6.7 Hz, -CH_2_), 4.60–2.78 (m, H_cellulose_, -C*H*_2_OH), 2.25 (t, *J* = 7.5 Hz, -CH_2_), 1.60 (m, -CH_2_), 1.33 (m, -CH_2_), 0.83 (m, -CH_2_CH_2_C*H*_3_).

^13^C NMR (101 MHz, Chloroform-*d*) δ 173.6 (C=O), 102.9–72.9 (C1-C5), 75.0 (-*C*H_2_CH_2_CH_3_), 64.2 (-*C*H2), 34.2 (-*C*H2), 28.4 (-*C*H2), 25.6 (-*C*H2), 24.6 (-*C*H2), 23.4 (-CH_2_*C*H_2_CH_3_), 10.7 (-CH_2_CH_2_*C*H_3_).

IR [cm^−1^] 3442, 2943, 2866, 1721, 1470, 1419, 1397, 1366, 1294, 1240, 1187, 1163, 1105, 1089, 1065, 1045, 961, 934, 840, 732, 710, 583.

M_n_ = 29.089 g mol^−1^, M_w_ = 49.851 g mol^−1^, D = 1.7

6-*O*-PLA-2,3-*di*-O-alkyl-cellulose

As with ε-caprolactone, the cellulose ethers were mixed with L-lactide using magnetic stirring, and heated to 110 °C in order to obtain a homogeneous melt, as described for the reactions with PCL. The polymerization was initiated by the addition of Sn(Oct)_2_ (0.05 eq), and the reaction mixture was stirred under argon for 16 h at 110 °C. After cooling to room temperature, the crude copolymer was dissolved in DCM and precipitated in hexane, washed with hexane and a hexane/toluene solution (20 mL/80 mL) by suspending the polymer in these solvents followed by decantation. The products were dried under reduced pressure to constant weight using a rotary evaporator.

6-*O*-PLA-2,3-*di*-O-benzyl-cellulose (**12**)

^1^H NMR (400 MHz, Chloroform-*d*) δ 7.40–6.97 (m, H_aromat_), 5.16 (m, -CH), 5.05–2.94 (m, H_cellulose_, -CH_2_), 1.52 (m, -CH_3_).

^13^C NMR (101 MHz, Chloroform-*d*) δ 169.7 (C=O), 139.0 (C_aromat_), 138.3 (C_aromat_), 128.3 (C_aromat_), 127.8 (C_aromat_), 102.7–64.2 (C_cellulose_), 69.1 (-CH), 16.7 (-CH_3_).

IR [cm^−1^] 3509, 2993, 2942, 1749, 1552, 1497, 1453, 1381, 1359, 1309, 1265, 1183, 1124, 1084, 1044, 914, 863, 737, 697, 620, 546.

M_n_ = 12.107 g mol^−1^, M_w_ = 15.556 g mol^−1^, D = 1.3

6-*O*-PLA-2,3-*di*-O-allyl-cellulose (**13**)

^1^H NMR (400 MHz, Chloroform-*d*) δ 5.94–5.65 (m, =CH), 5.35–5.03 (m, -CH), 4.47–3.91 (m, =CH_2_), 3.12 (m, H_cellulose_, -CH_2_), 1.65–1.40 (m, -CH_3_).

^13^C NMR (101 MHz, Chloroform-*d*) δ 169.7 (C=O), 135.6 (C=C), 134.7 (C=C), 116.8 (C=C), 116.1 (C=C), 103.1–64.0 (C_cellulose_) 74.0 (-*C*H_2_), 69.1 (-CH), 16.8 (-*C*H_3_).

IR [cm^−1^] 3492, 2993, 2941, 1749, 1453, 1382, 1358, 1264, 1182, 1127, 1084, 1044, 927, 867, 755, 699.

M_n_ = 13.155 g mol^−1^, M_w_ = 18.498 g mol^−1^, D = 1.4

6-*O*-PLA-2,3-*di*-O-propyl-cellulose (**14**)

^1^H NMR (400 MHz, Chloroform-*d*) δ 5.14 (q, *J* = 7.1 Hz, -C*H*), 4.62–2.68 (m, H_cellulose,_ -C*H*_2_CH_2_CH_3_), 1.65–1.40 (m, -CHC*H*_3,_ -CH_2_C*H*_2_CH_3_), 0.86 (m, -CH_2_CH_2_C*H*_3_).

^13^C NMR (101 MHz, Chloroform-*d*) δ 169.7 (C=O), 75.3 (-*C*H_2_CH_2_CH_3_), 69.09 (CH), 23.41(-CH_2_*C*H_2_CH_3_), 16.7 (-*C*H_3_), 10.7 (-CH_2_CH_2_*C*H_3_).

IR [cm^−1^] 3492, 2943, 1750, 1452, 1381, 1361, 1265, 1182, 1127, 1084, 1043, 952, 867, 755, 702.

M_n_ = 14.736 g mol^−1^, M_w_ = 22.883 g mol^−1^, D = 1.5

### 2.2. Fourier Transform Infrared Spectroscopy

Fourier-transform infrared (FTIR) spectra were measured (Nicolet 380, Thermo Fisher Scientific, Waltham, MA, USA) using attenuated total reflection (ATR). The spectra were measured between 4000 and 550 cm^−1^ with a resolution of 4 cm^−1^.

### 2.3. NMR Spectroscopy

Nuclear magnetic resonance spectra (NMR) were recorded at 100 MHz for ^13^C spectra and 400 MHz for ^1^H spectra (ECS-400 NMR, Joel, Akishima, Japan). The software MestReNova v12.0.0-20080 2017 Mestrelab Reasearch S.L was used for spectra analysis. Chemical shifts are expressed in ppm in respect to signals of deuterated solvent or TMS standard.

### 2.4. Size Exclusion Chromatography

Size exclusion chromatography (SEC) was utilized for molecular mass determination (PSS SECurity GPC system, pump PSS SECurity refractive index detector PSS SECurity RI, column PSS SDV 5 μm precolumn, PSS SDV 5 μm 100,000 Å, PSS SDV 5 μm 1000 Å). Chloroform was used as eluent at a flow rate of 0.7 mL min^−1^. The molecular weights were determined using a polystyrene calibration.

### 2.5. X-ray Diffraction

The data used in this work were obtained from a Bragg–Brentano powder diffractometer (XRD, Miniflex, Rigaku, Tokyo, Japan) with a copper anode, and a silicon strip detector (D/teX Ultra, Rigaku). The setup in detail: goniometer radius 150 mm; both Soller slits 2.5°; divergence slit fixed at 0.625°, but closing variably below 2*θ* = 10°; variable anti-scatter screen; no monochromator; kβ filter 0.06 mm nickel foil; effective receiving slit of the multiline detector 0.1 mm.

Samples were in the shape of platelets and had been stored for several days at room temperature. They were measured on low-background monocrystalline silicon holders, which were spun during measurements to reduce the effects of preferred in-plane orientation. The data was corrected for incoherent scattering from the holders through the subtraction of a blank measurement.

Data from samples with poly(ε-caprolactone) ligands was evaluated with Rietveld refinement (BGMN, using the Profex interface) [38,39], considering the machine line function, as verified by refining NIST Standards 640e and 660c (silicon and lanthanum hexaboride for peak shapes and positions; machine parameter file and refinement results available from the authors). The scattering angle range 7° < 2*θ* < 90° was considered, and the sample offset from the goniometer axis refined.

For the refinements, we used the structural model determined by Chatani et al. [40], and confirmed by Hu and Dorset [41]. As sample parameters, we refined the lattice parameters, the average crystallite size $\bar{L}$, the breadth of the microstrain distribution function $\varphi^{-1}\left(0\right)$ [42], and the mean squared atomic displacements that govern the attenuation of coherently scattered intensities into incoherent diffuse scattering. Preferred orientation was accounted for by a spherical harmonics function of the order *n* = 2.

We modeled the incoherent portion of scattered intensities based on a polynomial function, with the addition of two amorphous peaks around 2*θ* = 20°. The data from samples with poly(L-lactic acid) ligands lacked Bragg reflexes for a Rietveld refinement of the polymer phase.

The Rietveld-refined data was then used to determine the amount of crystalline phase $x_{c}$ using the method of Ruland and Vonk, with the lower limit of *s_p_* used to fit the polynomial extrapolation curve to *R*(*s_p_*) set to 0.5 Å^−1^ [43,44].

### 2.6. Differential Scanning Calorimetry

Differential scanning calorimetry (DSC) was performed with a DCS 1 Star^e^ (Mettler Toledo, Columbus, USA), using alumina crucibles and sample weights in the range of 7.0 and 11.0 mg.

For samples with PLA ligands, the temperature range from 240 K to 470 K was assessed; for samples with PCL ligands, the range was from 200 K to 420 K. For each sample, two cycles were measured in succession, with heating and cooling rates of 10 K/min, resulting in data of heat flow $\dot{Q}$ over temperature *T* and time *t*.

From the second measurement cycles, glass transition points $T_{g}$ were identified and determined as the temperature at the position of the maximum gradient of $\dot{Q}$ where the progressions of $\dot{Q}$ showed a distinct step from lower to higher values with *T*. For materials exhibiting $T_{g}$, the progressions of the constant–pressure heat capacities in their glassy and rubbery states $c_{p,g}$ and $c_{p,\;r}$ were approximated each using a median-based linear Theil-Sen estimator.

For both measurement cycles, melting and crystallization processes were identified as skewed peaks of $\dot{Q}$. Temperature values of identified melting points from the two heating cycles $T_{m,1/2}$ and the crystallization points $T_{c,c}$ from the cooling cycle were determined as the respective maximum absolute values of $\dot{Q}$. Their corresponding heats of fusion were determined as their integrals over the time axis, as shown in Equation (1). They were used to calculate values of crystallinity as their fraction of the reported heats of fusion for the respective crystalline phase, as presented in Equation (2).

(1) $$H_{f}=\int \left(\dot{Q}\left(t\right)-c_{p}T\left(t\right)\right)dt$$

(2) $$x_{c}=H_{f}/H_{f,c}$$

## 3. Results and Discussion

### 3.1. Syntheses

**Figure 1:**
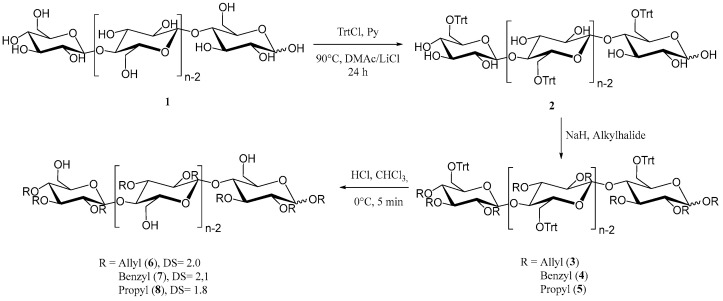
Synthesis of regioselectively substituted 2,3-di-O-alkyl cellulose derivatives as macroinitiators for the consecutive ring opening polymerization with ε-caprolactone.

To obtain the desired cellulose-based macroinitiators, initial tritylation provided the protected 6-*O*-trityl-cellulose (**2**) as depicted in Figure 1 [45]. The degree of substitution was determined to be 0.96 using ^1^H-NMR analysis. Hydroxyl groups in position 2 and 3 were then converted to the respective alkyl ethers (**3**, **4**, **5**) through reaction with either benzylbromide, allylbromide or propylbromide, similar to etherification reactions of cellulose reported in the literature [46]. To obtain unprotected primary hydroxyl groups for ring opening polymerization of ε-caprolactone and L-lactide, respectively, deprotection of the triphenyl methyl ether was carried out using gaseous hydrogen chloride to obtain the unprotected compounds **6**, **7** and **8**. All products were analyzed using infrared and NMR spectroscopy for structural elucidation in order to confirm our results (SI Figures S1 and S2). The degree of substitution was determined using ^1^H-NMR spectroscopy after peracetylation by comparing of integrated signals from the protons of the cellulose backbone and aromatic signals in the case of the benzyl derivative **7**, allylic protons for **6** and CH_2_ protons of the propyl chain in the case of compound **8 [18,47]**. The lack of signal splitting in the ^13^C-spectra also indicates satisfactory regioselective modification, with minor differences in DS that may occur, for example, due to differences in reactivity of the alkyl halides used in the reactions, as well as differences in the solubility of the products.

Infrared spectra show characteristic absorptions for each of the compounds. Absorption is at 1646 cm^−1^ (C=C) and 920 cm^−1^ (CH out of plane vibration) for allyl ether **6** and at 734 cm^−1^ and 696 cm^−1^ for the benzyl ether **7**. No absorptions for the trityl group at 632, 699, 747 and 767 cm^−1^ were observed after deprotection, which was further confirmed with NMR analysis. Aromatic signals were absent in all ^1^H spectra of deprotected substances with the exception of compound **7**. Here, as well as for compounds **6** and **8,** we could clearly determine the successful deprotection from the ^13^C spectra. The most distinguishable signals from the trityl moiety at 86.4 ppm (-*C*HPh_3_), as well as signals from quaternary aromatic carbon atoms at 143.4 ppm, were missing, which further confirms the synthesis of our desired cellulose-based macroinitiators.

**Figure 2:**
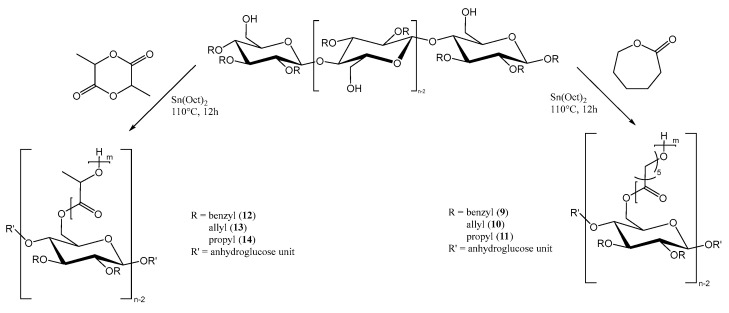
“Grafting from” polymerization of ε-caprolactone- or L-lactide monomers from functionalized cellulose macroinitiators.

For the preparation of the copolymers via ring opening polymerization (Figure 2), Sn(Oct)_2_ was used as catalyst, as previously reported for the synthesis of both homo- and copolymers of PLA or PCL. In our case, the hydroxyl groups at C6 of the anhydroglucose units had the function of initiating the ring opening polymerization.

**Figure 3:**
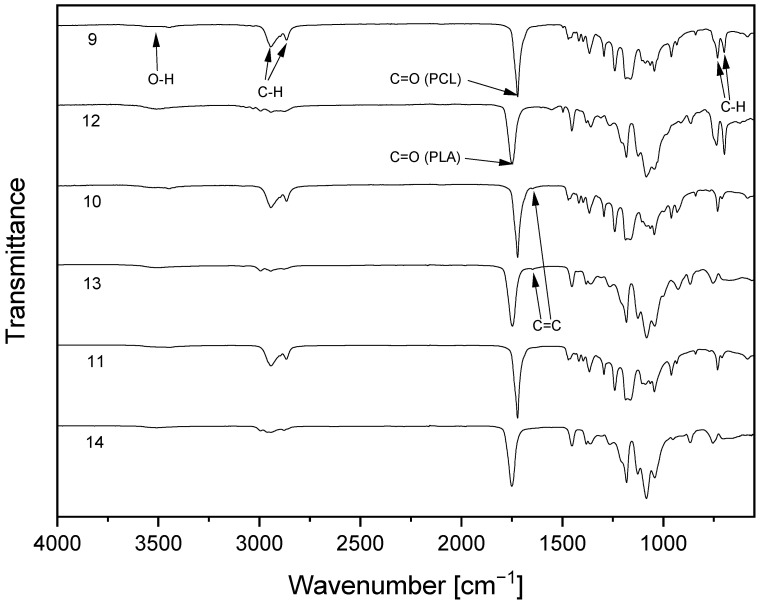
FTIR spectra of cellulose-based copolymers with poly(ε-caprolactone) (9–11) or poly(L-lactide) (12–14) sidechains.

The successful polymerization reactions were verified through FTIR of the resulting copolymers (Figure 3). Small absorption bands of cellulose or polyester hydroxyl groups are visible in the range of 3500cm^−1^ and C–H vibration in the range of 2900 cm^−1^. The infrared spectra of the copolymers also show absorptions at around 1720 cm^−1^ for PCL, and at 1748 cm^−1^ for PLA-copolymers, which is indicative of the C=O vibration of the ester group. In the case of compounds **9** and **10,** absorptions characteristic of aromatic groups are detected at 700 and 730 cm^−1^. This is a consequence of C–H vibrations due to the substitution of benzylic groups at position 2 and 3 of the cellulose backbone. For the allyl-substituted compounds **10** and **13**, an additional absorption at 1647 cm^−1^ can be observed. This is attributed to allylic double bond due to C=C stretching vibration of the unsaturated compound.

**Figure 4:**
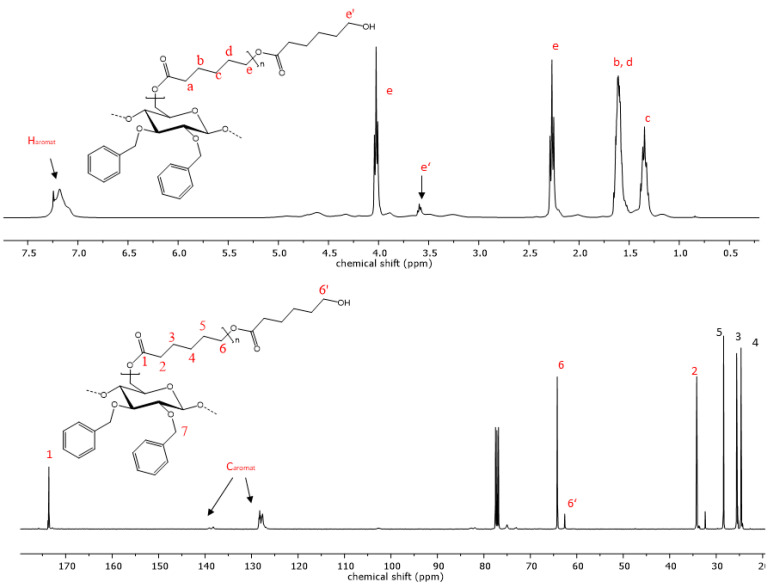
Representative ^1^H- and ^13^C-NMR spectrum of copolymer **9** with PCL sidechains.

**Figure 5:**
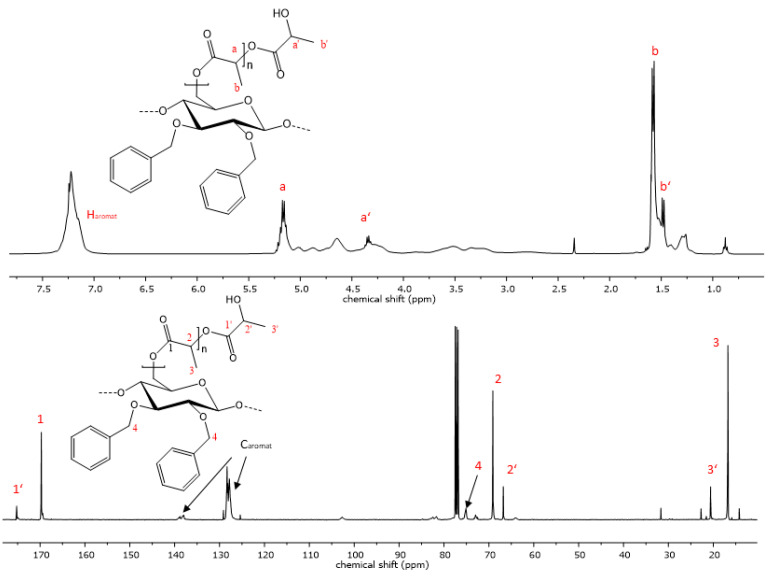
Representative ^1^H- and ^13^C-NMR spectrum of copolymer **12** with PLA sidechains.

The reaction progress was further analyzed using ^1^H- and ^13^C-NMR spectroscopy. The successful polymerization is clearly shown by characteristic signals of the polyester sidechains in the proton NMR-spectra. Signals that are characteristic of PCL are visible in the proton spectra. Triplets, which are representative of methylene groups in the vicinity to the carbonyl groups of the polyester, appear at chemical shifts of 4.02 and 2.27 ppm (Figure 4). Multiplets at 1.62 and 1.36 ppm are also due to -C*H*_2_- protons the PCL repeat unit, analogous to the respective homopolymers [48]. Additionally, the signal at 3.6 ppm is indicative of free hydroxyl groups at the chain end of the grafted polymer. The highfield shift of this methylene endgroup signal occurs due to the close proximity of the -C*H*_2_- protons to the hydroxyl function. In the case of PLA copolymers, the signal of the methyl group appears at 1.58 ppm and the methine signal is visible at 5.17 ppm (Figure 5) [49]. Aromatic signals from the benzyl group, which is attached at the C2 and C3 of the cellulose backbone, are visible at 7.20 ppm in both copolymers, while signals of the cellulose skeletal protons, as well as of the -C*H*_2_Ph group, are visible in the range of 5.14 and 3.00 ppm.

When the allyl- or propyl-modified compounds **6** and **8** were utilized as macroinitiators, additional signals of the unsaturated moiety or the ethyl group appear in the NMR spectra, respectively. The vinyl group protons appear at 5.80 and 5.12 ppm, and the methylene bridge protons at a chemical shift of 4.14 ppm and is overlapped by signals from the cellulose protons. The same applies for the methylene protons of the propyl derivative, whereas the signal of the methyl group is visible at a chemical shift of 0.86 ppm as expected.

The ^13^C-spectrum also shows characteristic signals of the PCL side chains. The signal at 173.6 ppm is characteristic of the quaternary carbon atom of the ester. Carbon signals of the PCL methylene groups can be observed at 64.2, 34.2, 28.4, 25.6 and 24.6 ppm. Signals of the cellulose skeletal carbon atoms are in the range of 102.8 to 64.2 ppm, while the signal of the C6 atom is superimposed by the signal from the polycaprolactone methylene endgroup. In the case of the PLA copolymers, the C6 signal becomes visible again at 64.1 ppm due to the downfield shift of the respective endgroup signal (Figure 5). Signals from the functionalization of the cellulose backbone prior to the polymerization reaction are also visible. Signals of aromatic carbon atoms of compounds **9** and **12** can be observed at 139.0 and 138 ppm, as well as at 128 ppm. The corresponding -*C*H_2_-peak of the benzylgroup is visible at 75.0 ppm. In the case of compound **10**, the vinyl carbons exhibit signals at 135.7 and 134.8, as well as at 116.5 and 115.9 ppm. Another signal from the methylene carbon is visible at 73.8 ppm. The graft copolymers derived from 2,3-di-*O*-propyl cellulose also shows peaks representative of the methylene groups at 75.0 and 23.4 ppm and an additional signal at 10.6 ppm as a result of the propyl-CH_3_ group.

### 3.2. Physical Properties

To determine the physical properties of our copolymers and to evaluate if, or to what degree, the presence of the cellulose backbone, and thereby the copolymer architecture, influences the properties of the poly(ε-caprolactone) and polylactic acid, XRD spectra (SI Figure S3) were recorded and differential scanning calorimetry (SI Figure S4) was performed. Compounds are named materials with PLC/PLA ligands and numbered according to Figure 4 for consistency: 9–11 for PCL and 12–14 for PLA.

X-ray diffractograms of materials with PLA ligands were dominated by a broad amorphous peak centered around 2*θ* = 19°. They showed some sharp reflexes of low intensity at 2*θ* = 13.4°, 23.2°, 31.7° and 45.4°, which could not be attributed.

On the other hand, materials with PCL ligands exhibited diffractograms dominated by Bragg reflexes of high intensity, most notably with three strong reflexes at 2*θ* = 21.4°, 22.0° and 23.7°. These corresponded to the {110}, {111} and {200} reflexes of the crystalline unit cell used for refinement. The structural characteristics of these materials, as determined with the Rietveld refinement, and using the method of Ruland and Vonk, are compiled in Table 1. The refined directional weighting factors *W_hkl_* indicate an in-plane preferred orientation of the {001} family of lattice planes, which corresponds to the direction of the molecular long axes in real space. This, in turn, can be attributed to the platelet shape of the measured materials. Nevertheless, sufficient intensities of {*hkl*} | *l* ≠ 0 reflexes were recorded for a satisfactory structural refinement.

Scanning calorimetric curves of materials with PLA ligands showed notable steps in the progression of $\dot{Q},$ indicative of a glass transition, but no melting or crystallization signals. Vice versa, in materials with PCL ligands, $\dot{Q}$ exhibited strong melting and crystallization signals on heating and cooling, respectively. However, it only showed a shallow gradient below 230 K on cooling and second heating. We interpret this as a gradual glass transition process but were unable to determine values of $c_{p,g}$ due to the lack of a discernable low-temperature plateau within the measured range. Hence, we only report values of heat capacity in the rubbery state $c_{p,r}$.

In Table 2, the approximated linear progressions of the *c_p_* were used to calculate their values at *T*_n_ = 298.15 K. For the determination of *x*_c_ with Equation (2), we used the value reported for poly(ε-caprolactone) of *H*_f,c_ = 139 J/g [50].

For materials with PLA ligands, the glass transition points *T*_g_ and heat capacities at normal temperature in the glassy state *c_p_*_,r,g_ determined in this work correspond roughly to those determined in the literature [51,52,53]. Their heat capacities in the rubbery state *c_p_*_,r,r_ match the expectation given by Equation (3) when applying the values of *f*_bb_ from Table 3, *c_p_*_,r,PLA_(*T*_n_) = 1.984 J (g K)^−1^, [52,53] $c_{p,\mathrm{cell}}^{M}\left(T_{n}\right)=227.0\;J\;{\left(\mathrm{mol}\;K\right)}^{-1}$ [54], and $c_{p,\mathrm{lig}}^{M}\left(T_{n}\right)=\left[103.7,\;63.8,\;73.6\right]\;J\;{\left(\mathrm{mol}\;K\right)}^{-1}$, for the ligands toluene [55], propene [56] and propane [57], respectively. *M*_cell_ = 162. 14 g mol^−1^ and *M*_lig_ = [92.14, 42.08, 44.10] g mol^−1^ denote the molar masses of glucose and of the ligands.

(3) $$c_{p,r}\left(T_{n}\right)=f_{\mathrm{bb}}c_{p,\mathrm{bb}}\left(T_{n}\right)+\left(1-f_{\mathrm{bb}}\right)c_{p,r,\mathrm{PLA}}\left(T_{n}\right)$$

(4) $$c_{p,\mathrm{bb}}\left(T_{n}\right)=\frac{c_{p,\mathrm{cell}}^{M}\left(T_{n}\right)+2c_{p,\mathrm{lig}}^{M}\left(T_{n}\right)}{M_{\mathrm{cell}}+2M_{\mathrm{lig}}}$$

This finding confirms the near quantitative etherification of the cellulose C2 and C3 hydroxyl groups, and the linear mixing of the heat capacities of the constituents of all the materials. The equilibrium melting temperature of PLA was reported to be $T_{m}^{0}\approx 480\;K$ [51,52]. Actually measured melting points are typically close to 450 K [58], being lowered due to the Gibbs–Thomson effect [52]. This would place them within the temperature range assessed in this work.

Hence, the lack of melting points determined using DSC in this work supports our findings with XRD, namely that the materials with PLA ligands consisted of exclusively amorphous atomic arrangements.

For pure PCL, values of *T*_g_ = 209 K were reported [59,60]. Our material with PCL ligands showed no discernable glass signals during DSC measurements. By comparison to other polyesters and polyamides [59], and specifically other polylactones [60], PCL exhibits a comparatively weak ∆*c_p_* on devitrification, which is further lowered by the amount of cellulose in the grafts, and by the amount of crystalline phase within the PCL portion.

For materials with PCL ligands, the values of *c_p,_*_r_(*T*_n_) could not be traced using Equation (3), when modeling $c_{p,\mathrm{lig}}^{M}\left(T_{n}\right)$ using linear mixing for the heat capacities of crystalline/glassy and amorphous PCL, $c_{p,g,\mathrm{lig}}=1.42\;J\;{\left(g\;K\right)}^{-1}$ and, $c_{p,r,\mathrm{lig}}=1.81\;J\;{\left(g\;K\right)}^{-1}\;$ [59,60], based on the values of crystallinity from Table 2 and Table 3. In all cases, the measured heat capacities are larger than those expected, which would be in the range of 1.62 J (g K)^−1^ < $c_{p,r}\left(T_{n}\right)$ < 1.68 J (g K)^−1^, for which we can currently offer no explanation.

In the materials with PCL ligands, we consider the properties of the crystalline phase: The higher values of *x*_c_ determined with XRD and with DSC on the first heating run, compared to those from the cooling and second heating run, point to the crystallization behavior typical of polymers, where post-crystallization takes place over several days, albeit at ever-diminishing rates. This is supported by the values of ${\bar{L}}_{100}$ and *φ*^−1^(0), obtained from aged materials. Both correlate negatively with *x*_c_, indicating a reduction in the average strain on volume elements of crystallites during their growth and maturation. In agreement, Benedict et al. found that pure PCL reached ”an equilibrium crystallinity [...] after several days incubation at room temperature” [61]. The values of *x*_c_ determined in the first DSC heating run are lower than those reported in the literature, where crystallinities larger than 0.6 were reported [61]. This is still true when considering the amounts of cellulosic backbone in the materials with PCL ligands (Table 3). We assume that the equilibrium crystallinities in PCL grafted onto cellulose are lowered due to steric hindrance caused by the graft backbones. The uniformly low values of ${\bar{L}}_{001}$ are indicative of a crystallite morphology consisting of self-folded chains that assemble into precursor laths, which in turn consolidate into single crystals by stacking along one of the directions perpendicular to the molecular chains, as described for the equally orthorhombic P2_1_2_1_2_1_ crystalline poly(3-hydroxybutyrate) [62]. Our results suggest that each fold has a width of 6 unit cells, corresponding to 12 repeating units of the molecular chain before turning into the opposite direction. This finding is at odds with those by Phillips at al., who found, based on optical microscopy [63] and via the Gibbs–Thomson equation [64] that the lamellar thicknesses in pure PCL were of the order of several 10 nm.

Additionally at odds are the melting temperatures reported in Table 3, which markedly lowered from the equilibrium $T_{m}^{0}$ ≈ 342 K [60,63] and are also lower than the $T_{m}$ ⪆ 330 K reported by Phillips et al. [64] To investigate, we used the Gibbs–Thomson Equation (5) as applied by Phillips et al. [64], together with their values for the end surface free energy $\sigma_{e}$ = 87 erg/cm^2^ and the heat of fusion *H*_f_ = 1.63 · 10^9^ erg/cm^3^ [63].

(5) $$L=\frac{2\sigma_{e}T_{m}^{0}}{H_{f}\left(T_{m}^{0}-T_{m}\right)}$$

Since the melting points reported in Table 3 represent the temperatures at maximum heat transfer, and the onsets of melting were ≈15 K lower, we inserted the values of *T*_m_,_1_ − 15 K into (5), thereby obtaining values of *L* ≈ 11 nm. This supports our finding that the dimensions of the primary crystallites are indeed ≈10 nm, as directly determined using XRD.

Given the lack of isolated intense {010} reflexes, the corresponding value of the average directional crystallite size ${\bar{L}}_{010}$ could not be determined with appreciable certainty. It is likely that, similarly to poly(3-hydroxybutyrate) [62,65], the stacking of precursor laths occurs preferably along either the direction ⟨100⟩ or ⟨010⟩, leading to extended shard-like crystallites.

## 4. Conclusions and Outlook

In this work, we were able to prepare graft copolymers of cellulose derivatives via regioselective modification of cellulose with consecutive ring opening polymerization of the ε-caprolactone or L-lactide monomers. The high emphasis on regioselectivity in our research thereby allowed the synthesis of high precision copolymers with an exact copolymer architecture, and through etherification, the implementation of functional groups onto the cellulose scaffold was possible while improving upon the material properties of native cellulose via consecutive ring opening polymerization. Structural analysis of the copolymers was performed using IR and NMR spectroscopy, as well as XRD and DSC measurements.

The prepared copolymers show great potential for further investigation concerning variation of the substituents at C2 and C3 of the cellulose backbone and post polymerization modification, either via functional groups at the cellulose hydroxyl groups, by endgroup modification of the polyester sidechains, or via the implementation of functionalized monomers for grafting polymerization. It is also possible to implement different polymers onto the cellulose backbone to obtain bottlebrush copolymers with two entirely different polymer segments through consecutive polymerizations, albeit adhering to a well-defined polymer architecture and simultaneously tailoring polymer properties in a targeted manner. The properties of such copolymers could then be exploited, e.g., for protein stabilization [11] as well as other bioconjugation applications using click chemistry [66].

## Figures and Tables

**Table 1:** Structural characteristics obtained using XRD: crystallinities xc, average crystallite sizes $\bar{L}$, microstrain distribution $\varphi^{-1}\left(0\right)$ and directional weighting factors W_hkl_.

Identifier	$x_{c}/1$	${\bar{L}}_{100}$$;\;{\bar{L}}_{010}$$;\;{\bar{L}}_{001}$/nm	$\varphi^{-1}\left(0\right)$/(mm/m)	[*W*_100_; *W*_010_; *W*_001_]/1
**9**	0.44	70; /; 9.9	9.1	0.40; 0.60; 0.00
**10**	0.47	41; /; 10.2	6.7	0.55; 0.45; 0.00
**11**	0.45	39; /; 11.3	8.7	0.59; 0.41; 0.00

**Table 2:** Structural characteristics obtained using DSC: glass transition points Tg, heat capacities in the glassy and rubbery states at Tn = 298.15 K, temperatures of melting and crystallization during the first heating, cooling and second heating Tm,1, Tc,c, Tm,2, and the corresponding amounts of crystalline phase $x_{c,1}$, $x_{c,c}$, $x_{c,2}$ calculated from the respective ∆Hf.

Identifier	*T*_g_/K	[*c_p,_*_g_; *c_p,_*_r_](*T*_n_)/(J (g K)^−1^)	[*T*_m,1_; *T*_c,c_; *T*_m,2_]/K	$[x_{c,1}$$,\;x_{c,c}$$,\;x_{c,2}]$/1
**9**	/	/; 1.93	326; 291; 320	0.34; 0.35; 0.34
**10**	/	/; 1.75	325; 278; 318	0.47; 0.33; 0.35
**11**	/	/; 1.77	324; 278; 317	0.42; 0.29; 0.33
**12**	319	1.36; 1.75	/; /; /	/; /; /
**13**	310	1.41; 1.86	/; /; /	/; /; /
**14**	318	1.31; 1.81	/; /; /	/; /; /

**Table 3:** Structural characteristics obtained using SEC: Mass fraction of the cellulosic backbones *f*_bb_.

Copolymer	*f*_bb_/(g g^−1^)
**9**	0.19
**10**	0.13
**11**	0.14
**12**	0.30
**13**	0.24
**14**	0.28

## Data Availability

The data presented in this study are available on reasonable request from the corresponding author.

## Supplementary Materials

The following supporting information can be downloaded at: https://www.mdpi.com/article/10.3390/polym15020455/s1, Figure S1: Fourier-transform infrared spectra of 6-O-trityl-protected cellulose derivatives; Figure S2: FTIR spectra of macroinitiators **7**, **8** and **9**; Figure S3: DSC traces of compounds **9** (a), **10** (b), **11** (c), **12** (d), **13** (e), **14** (f); Figure S4: X-ray diffractograms of the compounds **9** (a), **10** (b), **11** (c), **12** (d), **13** (e), **14** (f).
