# Supplementary material for: Synthesis and Characterization of Functional Cellulose–Ether-Based PCL- and PLA-Grafts-Copolymers
Source: Polymers (Basel). 2023 Jan 15;15(2):455. doi: 10.3390/polym15020455 (PMC9861352; doi:10.3390/polym15020455)
Supplement: Supplementary file 1 [file polymers-15-00455-s001.zip › polymers-2142054-supplementary.pdf]

## Supporting Information

# Synthesis and Characterization of Functional Cellulose–Ether-Based PCL- and PLA-Grafts-Copolymers

**Korbinian Sommer, Daniel Van Opdenbosch and Cordt Zollfrank ***

Chair for Biogenic Polymers, TUM Campus Straubing for Biotechnology and Sustainability, Technical University of Munich, 94315 Straubing, Germany
* Correspondence: cordt.zollfrank@tum.de

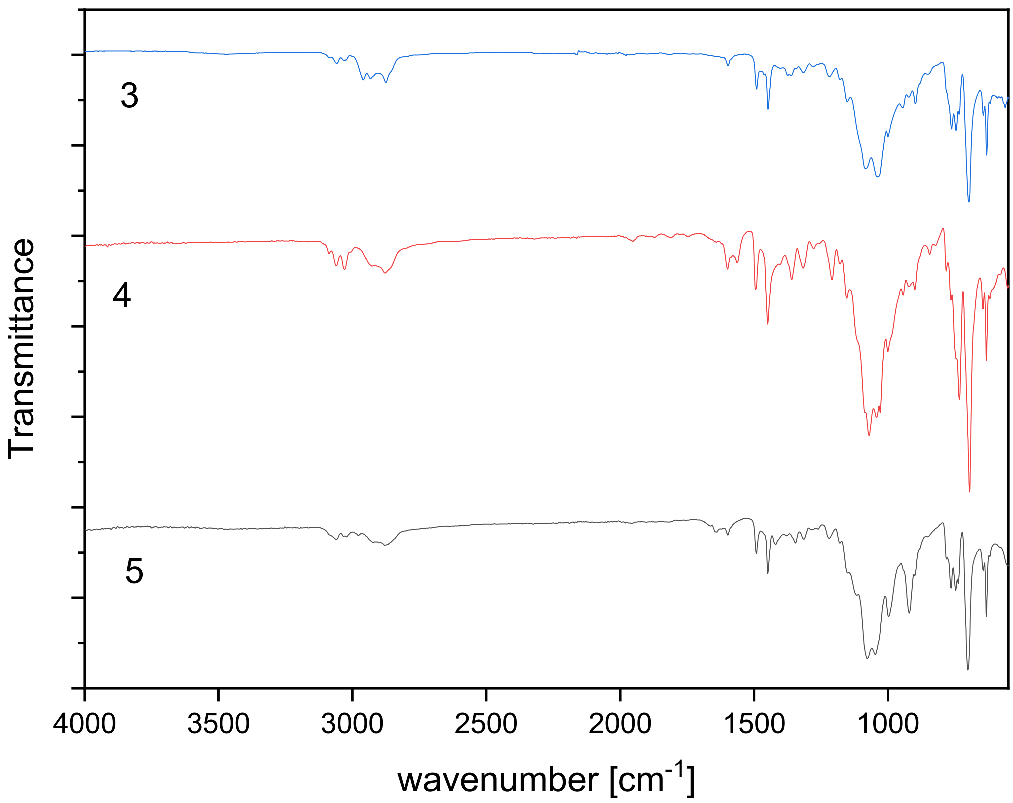


**Figure S1.** Fourier transform infrared spectra of 6-O-trityl-protected cellulose derivatives.

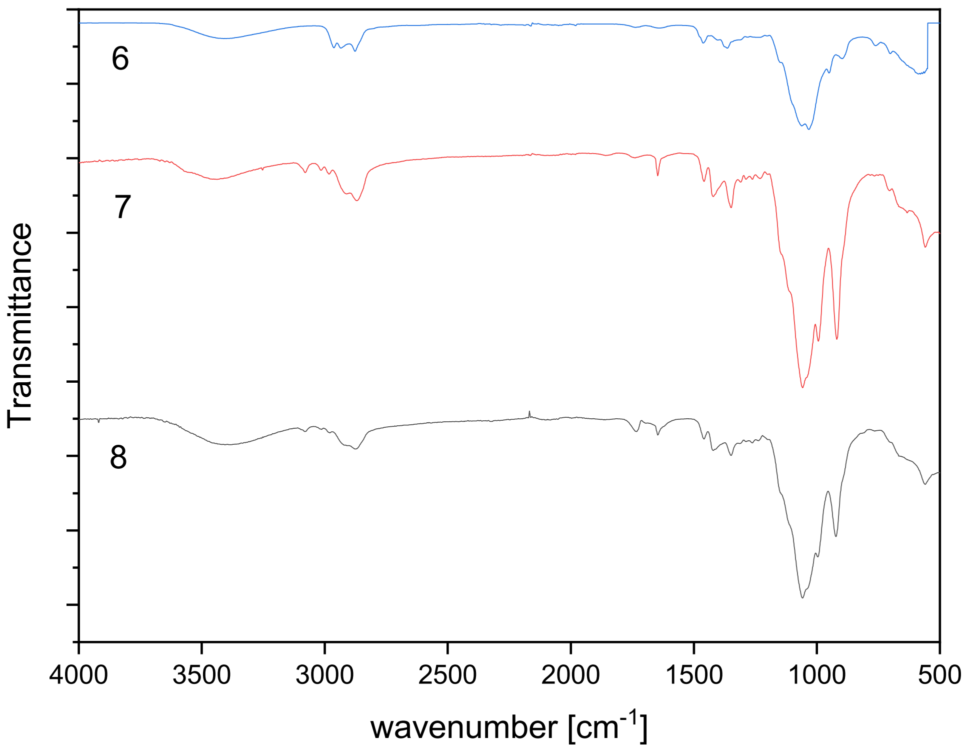


**Figure S2.** FTIR-spectra of macroinitiators **6**, **7** and **8.**

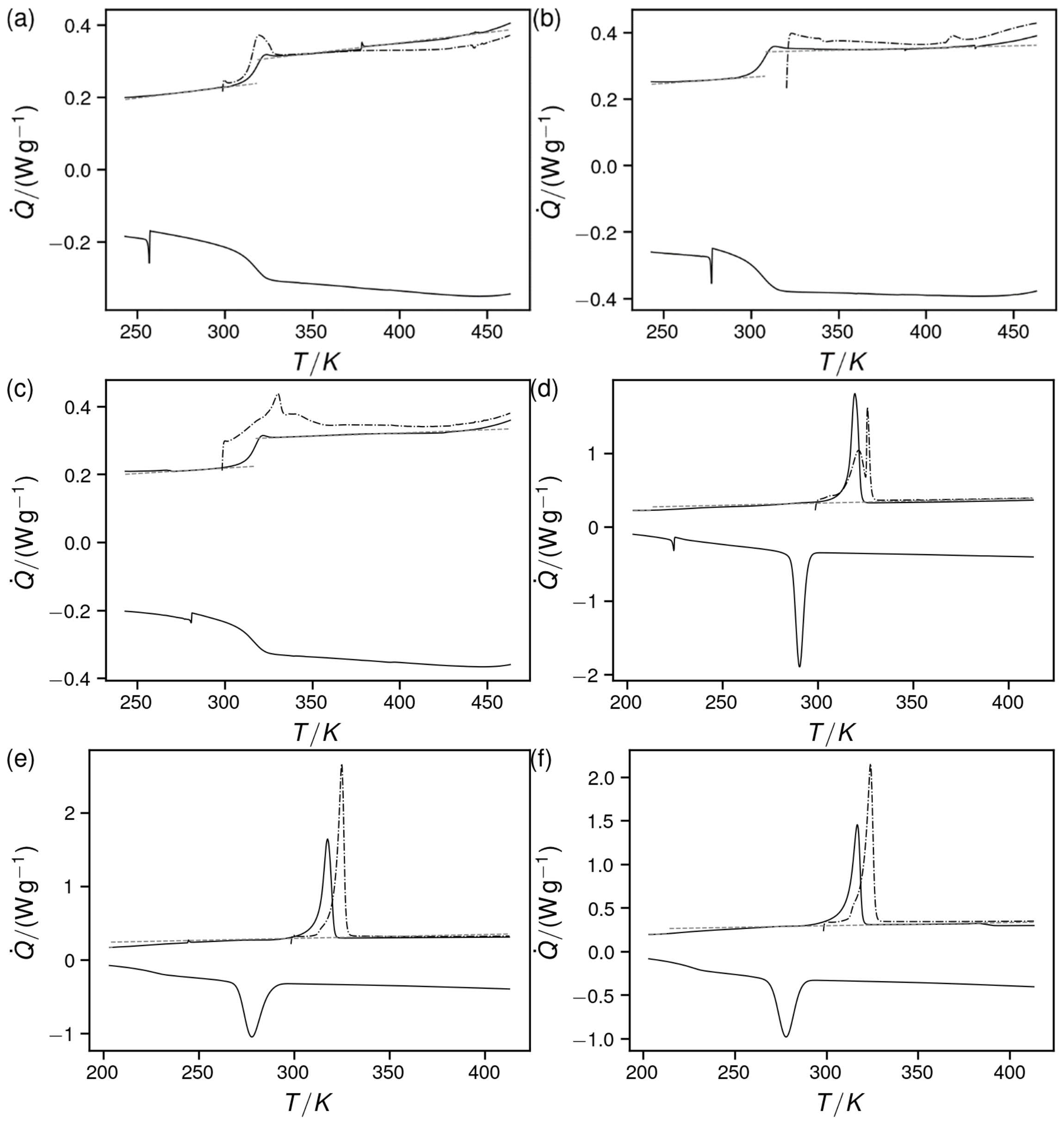


**Figure S3.** DSC traces of compounds 9 (a), 10 (b), 11 (c), 12 (d), 13 (e), 14 (f).

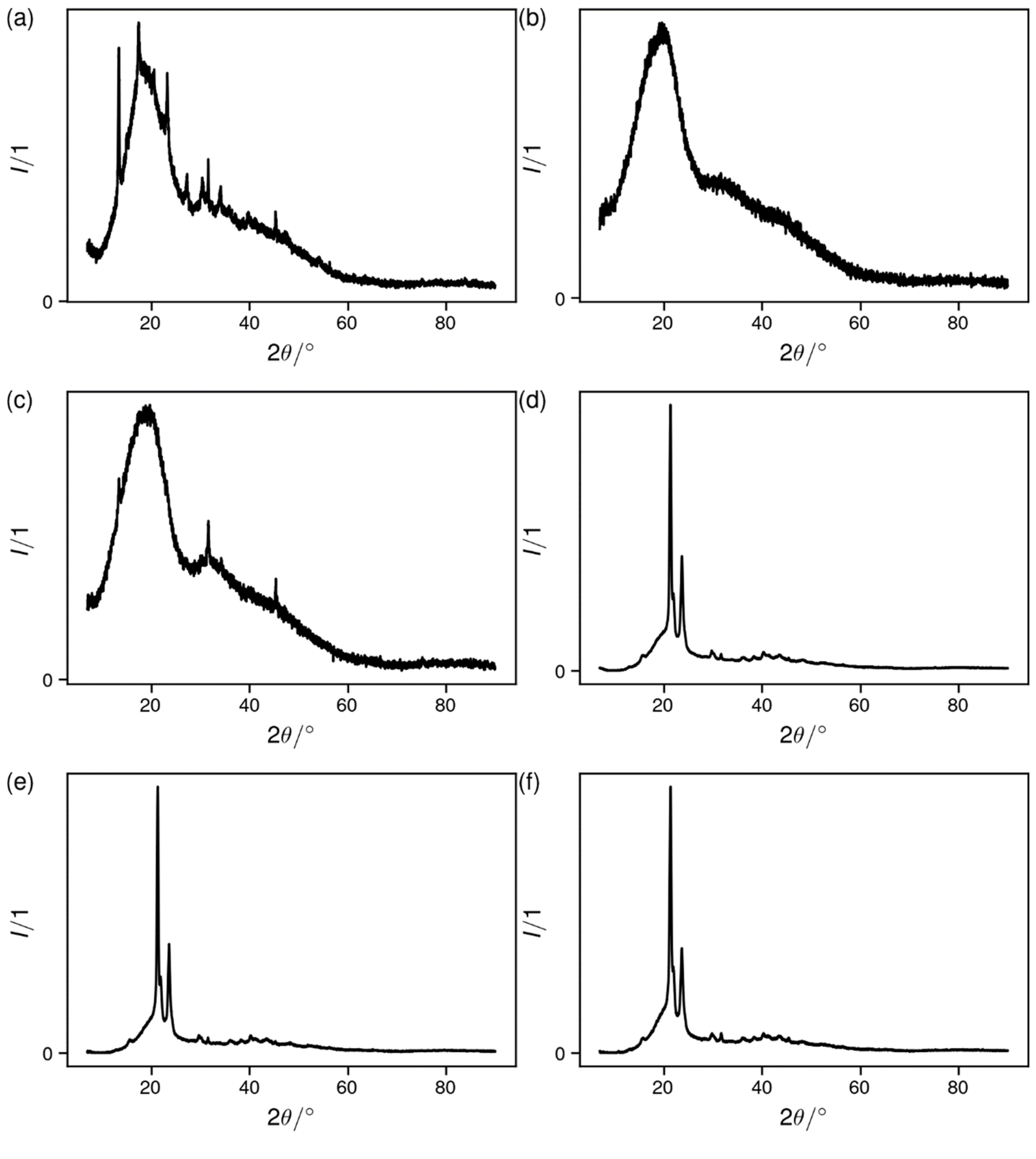


**Figure S4**. X-ray diffractograms of the compounds 9 (a), 10 (b), 11 (c), 12 (d), 13 (e), 14 (f).
